# Vegetable Consumption and Factors Associated with Increased Intake among College Students: A Scoping Review of the Last 10 Years

**DOI:** 10.3390/nu11071634

**Published:** 2019-07-17

**Authors:** Vanessa Mello Rodrigues, Jeffery Bray, Ana Carolina Fernandes, Greyce Luci Bernardo, Heather Hartwell, Suellen Secchi Martinelli, Paula Lazzarin Uggioni, Suzi Barletto Cavalli, Rossana Pacheco da Costa Proença

**Affiliations:** 1 Nutrition in Foodservice Research Centre (NUPPRE—Núcleo de Pesquisa de Nutrição em Produção de Refeições), Nutrition Postgraduate Programme (PPGN—Programa de Pós-graduação em Nutrição), Nutrition Department (Departamento de Nutrição), Campus Universitário João Davi Ferreira Lima, Federal University of Santa Catarina (UFSC—Universidade Federal de Santa Catarina), Florianópolis 88040-900, Brazil; 2 The Foodservice and Applied Nutrition Research Group, Faculty of Management, Talbot Campus, Bournemouth University, Poole BH12 5BB, UK

**Keywords:** young adults, nutrition guidelines, university, dietary needs, food behaviour

## Abstract

Vegetable consumption is a predictor for improved health outcomes, such as reduced obesity and likelihood of food-related noncommunicable diseases. Young adults are a key population, being in a transitional stage-of-life: Habits gained here are taken through the lifespan. This review establishes insight into the consumption of vegetables among young adults during their college/university years, and factors associated with increased consumption. Seventy-one papers were extracted, published between January 2009 and October 2018. Search terms related to consumption; vegetables; and college/university setting and sample. A diverse range of definitions, guidelines, and study approaches were observed. Findings identify that the majority of students do not consume World Health Organization recommendations. Being female was the most frequent predictor of higher intake of vegetables, and no consumption patterns were identified by countries. Living at family home; body mass index; happiness and stress level; perceived importance of healthy eating; socioeconomic level; breakfast consumption; stage of study; openness to new experiences; sleep pattern; nutrition knowledge; activity level; alcohol usage; and energy intake were identified as influential factors. Public policies and new strategies to encourage vegetable consumption among college students are indispensable, especially targeting subgroups with even lower intakes, such as males and those living outside family home.

## 1. Introduction

Young adulthood is a particularly important time for the promotion of healthy eating, because several behaviours are developed and established during this period [1]. However, as characterised as a transitional life stage which may include many significant changes, such as leaving the family home, commencing college, entering the workforce, partnering, or becoming a parent, many people lack interest in following a healthy and balanced diet, or struggle to prioritise this [2,3]. 

Young adults include people from different backgrounds in a relatively large age range, and the great majority of college students are part of this group. They are beginning to take responsibility for their own dietary habits as they undergo a critical period in the consolidation of eating habits and behaviours [4]. A review study demonstrated that most college students have unhealthy eating behaviours, including high intake of fast foods, snacks, sweets, soft drinks and alcoholic beverages, and low intake of fruits, vegetables, fish, whole grains and legumes [5].

The frequent poor dietary behaviours among young adults are among the key factors contributing to a weight gain trajectory and increased risk of noncommunicable diseases (NCDs), such as heart disease, cancer and diabetes (type 2) [6]. NCDs are estimated to cause 41 million deaths each year, equivalent to 71% of all deaths globally [7]. According to the World Health Organization (WHO) [8], the risk of developing NCDs could be reduced through an intake of ≥400 g of fruits and vegetables per day, which would also help to ensure an adequate daily ingestion of dietary fibre. Despite initiatives designed to increase fruit and vegetable intake, people across the globe struggle to meet this recommendation [9,10]. 

While the health benefits of a high fruit and vegetable consumption are well known [11], and considerable work has attempted to improve intakes [12], increasing evidence also recognises a distinction between fruit and vegetables, both in their impacts on health and in consumption patterns. A recent review suggests enhanced health benefits from a high consumption specifically of vegetables due to their protein and fibre content, yet intakes remain low [13]. Additionally, studies have highlighted ethical, environmental and cost advantages to diets with a higher vegetable composition [14]. Notwithstanding, evidence demonstrates that the intake of fruits tends to be higher due to their sweet taste, softer texture and easier manner of eating (usually raw and as a snack or dessert) [15]. A systematic review demonstrated that interventions to increase fruit and vegetable intakes more often target fruit and typically report greater success in fruit consumption compared to that of vegetables. Even so, few studies on food choice and eating behaviour have investigated vegetable intake as a separate variable. This is an important limitation of existing knowledge, since the factors that influence fruit consumption may not be applicable to vegetables. The majority of interventions aiming to increase the intake of vegetables as a separate and distinct food group have focused on younger children [16].

This review identifies and summarises previously published research on vegetable consumption among college students, focusing specifically on vegetables as a distinct food group. The objective of this scoping review is to establish insight into the consumption of vegetables (portions, grams, frequency; measured or reported) among young adults during the college/university years. Any factors associated with increased consumption of vegetables were retrieved and considered. An improved understanding of the factors that affect vegetable consumption is essential to improving the diet quality of populations. This paper makes an original and valuable contribution to existing knowledge that all too often has aggregated fruit and vegetable consumption and thus may have biased the key factors related to increased vegetable intake.

## 2. Materials and Methods

This scoping review included quantitative data from observational studies published between January 2009 and October 2018 investigating vegetable consumption among young adults in a college setting. Papers were included if vegetable consumption was assessed as a primary focus or part of a diet where data on vegetable consumption could be analysed separately. Data were evaluated for significance to determine which factors are associated with increased vegetable consumption among the targeted group. 

This study adopted an effective bibliographic research strategy aimed at reducing bias in the selection of articles for review. A literature search was conducted in October 2018 in the following databases: Scopus, MEDLINE/PubMed (via National Library of Medicine) and Scientific Electronic Library Online (SciELO). An additional search using the snowball method was performed, scrutinising the references in the review studies obtained from the initial search to ensure a comprehensive data collection. 

The terms used in the search comprised four categories that were combined using the Boolean operator “AND” as follows: (a) consumption (food consumption OR food intake OR eating), (b) type of food (vegetable), (c) setting (college OR university OR “higher education” OR faculty) and (d) sample (student OR freshman OR sophomore OR young adult OR millennial OR late adolesc* OR emerging adult* OR “new adult”). The combinations were adapted to use more general or more specific terms based on the limitations of each database. For the Scopus and Scielo databases, the search was performed considering the title, abstract and keywords, while in Medline/Pubmed, the terms were searched in the full text due to the low number of references (*n* = 20) when searching just title and abstract. Preliminary searches were helpful for adjusting search terms and their combination in order to find the largest possible number of articles related to the topic.

Studies published in Portuguese, Spanish and English were included. This was possible due to the international team of authors which enabled full assessment of studies published in these three languages. It was felt that inclusion of the widest possible range of studies enhances the value of the review, representing findings from a wide diversity of cultures and settings. Exclusion criteria comprised qualitative studies; studies focusing on eating disorders (e.g., bulimia) or specific groups (e.g., athletes, pregnant women), biomarkers and supplementation; studies with patients (e.g., menopause women, anaemics, people with coeliac disease); specific minerals association with vegetable consumption; validation and reliability of questionnaires; hypothetical scenarios and case-control studies. Intervention studies were not included to ensure that this review focused on establishing a base line account and to avoid repetition of the recent systematic review by Appleton et al. [16]. 

**Figure 1:**
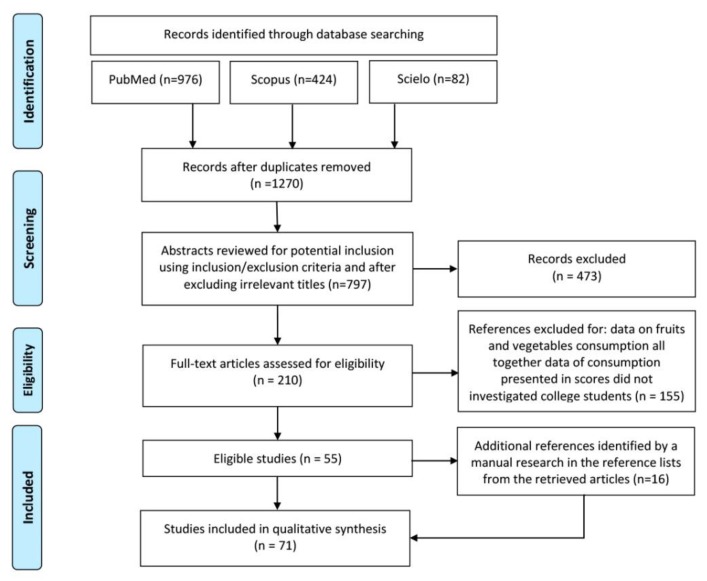
Identification and selection of articles for inclusion in this review.

Duplicates were removed, followed by irrelevant titles. The abstracts of the remaining papers were reviewed, and potential studies were considered based on the inclusion/exclusion criteria. The studies which analysed the intake of food or food groups, or the dietary patterns of college students were read and judiciously analysed in full text. Studies were not considered if they presented data on aggregated fruit and vegetable consumption; if they presented data of consumption in scores where it was impossible to estimate the consumption; if they were developed in the college setting with a different population (e.g., lecturers); and if they were not developed with college students. Figure 1 shows how the database search and article selection process resulted in 71 articles being included in this review.

The data of selected studies were extracted to a Microsoft Excel spreadsheet for analysis, including study details (i.e., authors, location, year of publication, and design), study population, sample and participant demographics, food intake assessment instruments, definition of vegetables, data on vegetable consumption and associated factors with increased vegetable intake. The information extracted from each study is presented in the summary tables. The percentage of male and female participants and mean age across all studies was calculated. The common results were grouped and presented separately according to the type of data provided (i.e., frequency of overall intake, frequency of intake according to portions/servings, average intake of portions/servings per week, consumption in grams/day and comparison of consumption with relevant guidelines). Mean daily vegetable intake was calculated across studies presenting the consumption of vegetables in frequencies of intake. A few studies are presented in more than one table.

Details from all studies were tabulated by one review author (VR) and checked by AF or GB. Tables are provided in the Results section. Tables outline vegetable consumption organised by frequency, quantity and comparison against relevant guidelines.

## 3. Results

### 3.1. Studies Characteristics

#### 3.1.1. Design and Participants

This study reviewed data from 71 articles regarding the vegetable consumption of 65,971 college students from more than 155 different colleges located in 30 countries from Africa (2), Asia (8), Europe (13), North (2) and South America (4) and Oceania (1). The majority of participants were female (69.8%), and the mean age of the students was 21.6 years old. Almost 95% of the studies (n = 67) were designed as cross-sectional. The other designs were mixed (cross-sectional and longitudinal) [17], microlongitudinal (21 days) [18], time series analysis [19], and retrospective survey [20].

The majority (70.4%) of the studies focused on evaluating elements of the whole diet of participants. Thirteen studies were specific regarding the consumption of fruits and vegetables [20,21,22,23,24,25,26,27,28,29,30,31,32], while only three studies were exclusively focused on vegetables [33,34,35]. Five studies investigated adherence to the Mediterranean diet and its relation with the consumption of specific food categories, such as vegetables [36,37,38,39,40]. 

The most usual instrument for assessing food consumption was the food frequency questionnaire (FFQ), used by twenty-eight studies [19,21,23,27,32,34,37,40,41,42,43,44,45,46,47,48,49,50,51,52,53,54,55,56,57,58,59,60]. Two of them combined the FFQ with 24-h recalls [27,59]. Another study also chose 24-h recalls [61] for assessing food consumption, while three studies used diet story questionnaires [35,62,63]. Thirty-one studies declared having used questionnaires, adapted or designed specifically for the study purposes [20,24,25,26,28,29,30,31,33,36,38,64,65,66,67,68,69,70,71,72,73,74,75,76,77,78,79,80,81,82,83]. Seven studies used prospective methods for evaluating food consumption [17,18,19,22,39,84,85]. The two studies in New Zealand [18,22] required participants to fill in 21-day food diaries. The time series analysis study [22] also evaluated a second sample, which was required to complete a 13-day food diary. Another three studies [17,19,39] employed a 7-day food record, one of them combined with a FFQ [19], and two studies [84,85] a 3-day food record.

#### 3.1.2. Vegetable Definition

Most studies (60.6%) did not define what was being considered as vegetables in their investigations. The term was only presented in the tables or text referring to the group, without specifying whether participants were told what to consider as a vegetable, or whether different types of vegetables consumed were grouped in this category. From the studies which mentioned what was considered in the analysis, eight divided vegetables into raw or fresh vegetables (including salads), and cooked vegetables [37,43,46,47,49,53,56,72]. Another six studies [19,34,36,38,44,58] declared that the intake of both raw and cooked vegetables was considered to calculate “vegetable consumption”. One study [45] only considered salad and raw vegetables in their analysis. Four studies divided vegetables into different categories: green, yellow, other vegetables, and salads [60], sautéed leafy greens, leafy greens, nonleafy cooked vegetables and nonleafy raw vegetables [21]; fresh, frozen, canned and stewed [22]; and fresh, tinned, legumes and potatoes [29]. Finally, eight studies included different forms of vegetables to create a single variable in their analyses: green-, red- or yellow-coloured vegetables [64]; vegetables without tubers, roots and bananas [48], fresh, canned or juice [25]; vegetables and juices [85]; raw, cooked, canned or frozen [77]; coloured and other types of vegetables, mushrooms and sea vegetables [63]; fresh, cooked or frozen, as well as green salad and did not count potatoes [31]; and vegetable side dishes or salads [32]. It is clear that the complexity of defining vegetables either by botanical or culinary descriptors makes it difficult to provide an aggregated analysis.

No study mentioned the degree of food processing related to the vegetables consumed i.e., whether the vegetables were fresh, minimally processed (e.g., washed, sliced, peeled), juiced, or preserved in brine or sugar. Additionally, there was no discussion regarding the type of production of these vegetables (organic or conventional), if they were originated from genetic modified crops, or the type of commercialization, for instance, whether they were part of fair trades, locally produced, or imported from other countries. 

#### 3.1.3. Vegetable Consumption

Vegetable consumption is summarised in five tables, according to the type of outcome measure provided in the studies. In the first table, data from 30 studies are presented with the frequency of vegetable intake (Table 1). Mean frequency of daily vegetable intake was 40.2%, varying from 11.2% to 72.4%. The highest frequency of daily intake of vegetables was observed in Finland, where 72.4% of females and 57.3% of males eat vegetables daily [45]. In a study conducted only with female participants in Poland, 65.0% ate salad and raw vegetables every day [75]. In Cyprus [38], 56.5% of the participants ate fresh or cooked vegetables daily and, from these, 29.5% more than once a day. Other studies also demonstrated a high frequency of intake, such as in Lithuania [49], where 60.0% eat vegetables 4–7 times a week, and in Italy [82], where 42.1% of participants ate vegetables at least once a day, and 16% in a frequency of 5–6 times a week.

On the other hand, some studies demonstrated frequencies of daily intake as low as 11.2% in Saudi Arabia [64], 12.4% in South Africa [59] and 14.3% in Zimbabwe [55]. Brazilian studies showed the lowest frequencies of vegetable intake. In Perez et al.’s [56] study, 28.4% and 25.5% of college students answered never eating raw vegetables/salads and cooked vegetables, respectively. In Cansian et al.’s [21] study, 25.2% of participants answered never or rarely eating sautéed leafy greens. Associated factors with increased intake were being female [36,49], regular health self-rate [79], lower BMI and lower blood pressure (both genders) [84], in later years of study [72], not being a quota student (an affirmative action approved by Law which reserves 50% of spots in Brazil’s federal universities for students coming from public schools, low-income families and who are of African or indigenous descent) [56], the importance given for eating healthy [47], and living at family home [44]. 

Table 2 presents frequency of vegetable consumption according to the portions/servings consumed. The most common frequency of intake was 1 portion/day. This level of consumption was achieved by 51.6% of Iranian students [25], 44.0% of Indian students [65] and 35.8% of Chilean [66]. The form of presenting the results was not uniform, and sometimes, only a percentage related to the consumption of a determined amount was presented, without specifying the distribution of the remaining percentage of consumption among students. For instance, the study of Duran-Aguero et al. in 2014 [67] presented that 21.8% of the investigated students consumed 2 portions of vegetables a day, while their study of 2016 [69] compared the percentage of normal and overweight/obese students who consumed 2 portions of vegetables a day, which was 32.4% and 43.9%, respectively. Moreover, a Saudi Arabian study [23] demonstrated that almost two thirds of nutrition department students (64.3%) consumed ≥3 servings of vegetables/day, while the non-nutrition department students consumed a lower percentage (45.5%). The studies showed that consuming two or more servings of vegetables was a protective factor for overweight/obesity [66] and that the measure of happiness was positively associated with the amount of vegetables consumed [25]. A higher frequency of vegetable intake was associated with both a higher frequency of eating episodes and a regular breakfast habit, and this association with breakfast habits is stronger for males than for females, while the association with the number of eating episodes was similar between sexes. A higher socioeconomic status and the intention to lose weight represented independent factors associated with more favourable vegetable consumption [28]. Students who lived in the family home consumed more helpings of vegetables each day, compared with young adults who lived independently [78]. 

In Table 3, the studies present the average portions/servings of vegetables consumed overall or by groups. The highest average intake of vegetables was identified in New Zealand and Canada. In New Zealand, Conner et al. [22] found frequencies of 2.5 servings/day in sample 1 and 2.8 servings/day in sample 2, while the microlongitudinal study developed by White et al. [18] found an average intake of 2.5 servings/day. In Canada, the regular intakes were 2.5 servings/day in 2010 [77] and 2.7 servings/day in 2013 [40]. Italian students living in the family home consumed higher quantities of cooked vegetables, whilst those living away from home were characterised by higher consumption of raw vegetables [37]. Italian women and students living at family home were positively associated with a greater consumption of vegetables [53]. In New Zealand, openness to new experience was the most consistent significant predictor of higher vegetable consumption. Young adults higher in openness ate more daily servings of vegetables than young adults lower in openness across both samples tested [22]. In Croatia, nutrition knowledge was significantly positively correlated with intake of vegetables [52], and women, senior students and those who prepare food for themselves demonstrated higher nutrition knowledge scores. In Italy, intention significantly affects vegetable eating behaviour in participants with low habits, while perceived behavioural control is the main predictor of the behaviour in the high habits group. This indicates that vegetable consumption may be intentional as well as habitual, depending on the level of habit strength [33]. Finally, in Spain, overweight people consumed significantly fewer vegetables than the normal weight ones. Females ate more raw or cooked vegetables than men [54].

A point to be considered in results presented in Table 2 and Table 3 is that not all the studies mentioned the equivalence in grams for the portions evaluated or whether participants were told what to consider as a portion.

In Table 4, studies are summarised according to vegetable consumption in grams/day. The higher average intake in grams per day was found in Iran [42] (263 g/day), followed by Japan (217.5 g/days) [35]. The lowest intake was observed in first-year students in Croatia (80 g/days) [86]. In Iran, compared with those in the lowest tertile, women in the top tertile of dietary energy density had the lowest diversity score for vegetables [41], and breakfast consumers had a larger intake of vegetables and higher scores for the dietary diversity score for vegetables (1.6 versus 1.2) [42]. In Japan, late midpoint of sleep was significantly negatively associated with the energy-adjusted intake of vegetables [63]. Additionally, in a Dutch study, vegetable intake was lower among students who were non-Dutch, living in the family home, not adhering to physical activity guidelines and moderate and heavy alcohol drinkers [32].

Table 5 presents studies comparing the vegetable consumption with relevant guidelines or recommendations that varied from daily [47] to five portions of vegetables per day [48]. The median frequency of participants who achieved relevant recommended vegetable intake was 35.4% but varied widely. The lowest frequency of compliance with recommendations was found in South Africa, where only 2.5% of participants met the recommendation of 3 portions/day [59], followed by two studies in the USA, in which 7.0% [85] and 12.4% [71] of the participants met the recommended 2.5 cups/day. The highest frequency (74.0%) was found among fourth-year students from the Netherlands (150 g/day) [17], followed by 68.4% for salad/raw vegetable intake (daily or several times a day) in a study in Finland [47]. Associated factors with meeting the relevant recommendations of vegetable intake were being female [46,47,54,79], importance given for eating healthy [47], normal weight [33,68] and less stress [46].

## 4. Discussion

This study presents worldwide data regarding vegetable consumption from almost 70 thousand college students. The findings demonstrate that the majority of young adults do not consume vegetables as frequently as recommended by the WHO, nor in sufficient quantities to satisfy other relevant guidelines. No consumption patterns according to country or region were apparent. Being female was the most frequent predictor associated with higher intake of vegetables [36,46,47,49,53,54,80]. The variation between genders was highlighted and might explain the large disparity in consumption among studies. For example, the highest consumption percentages were found in studies only with women or in studies with more than 70% of female respondents, such as Finland [45], Poland [75] and Spain [54]. This finding is also consistent with previous research showing that females eat healthier than males, as, for instance, male young adults eat more frequently at fast-food restaurants than female young adults [88], and male college students consume fewer servings of fruit and vegetables daily than female (4.3 vs 4.8; *p* < 0.05) [89]. In addition to this main predictor, the following factors were associated with higher intake of vegetables: normal weight [33,54,66,68]; living in the family home [37,44,53,78]; greater perception of happiness and less pressure and stress [18,25,43,46]; importance given for healthy eating [33,47,79]; higher socioeconomic level [28,56]; having breakfast [28,42]; lower BMI and lower blood pressure [84]; later stage of study [72]; more openness to new experiences [22]; early mid-point of sleep [63]; nutrition knowledge [52]; being more active and drinking less alcohol [32]; and lower energy diet density [41].

Studies on vegetable consumption often indicate health benefits from high consumption, such as reduced risk of cardiovascular disease, diabetes type 2, various cancers, stroke, dementia and cognitive decline [8]. The majority of studies, however, do not investigate vegetable consumption independent of fruit consumption or other aspects of the diet. While fruits and vegetables are frequently consumed together, associations may reflect not just the relationship with vegetables but with product consumption in general, or with a healthier diet/lifestyle [16]. Despite the study focus being the intake of vegetables in particular, the majority of data came from studies evaluating the overall diet intake of students. This might have hampered the analysis, considering that often only partial information regarding the consumption was available. Additionally, comparison between studies is problematic due to differing study approaches (e.g., grams per day; frequency of intake). In order to minimise this limitation, the results were grouped by type of data available. 

There was a lack of definition for vegetables in many studies, and many times, it was not possible to identify if any definition was given to participants at the point of research. In a few studies, the authors only used the term ‘vegetables’ to refer to the category. Other studies divided vegetables into ‘cooked’ and ‘raw/salads’, and a few were specific, dividing them, for instance, in ‘green’, ‘yellow’, ‘salad’ and ‘other vegetables’. Moreover, potatoes were sometimes included in the category and sometimes not considered at all. The authors believe that this lack of definition is a limitation in the study of vegetable consumption. 

Many issues were identified as challenges in the design, conduct, measurement, evaluation and comparison between studies on vegetable intake. Previous studies have highlighted that the term ‘vegetables’ covers a heterogeneous group of foods, especially across cultures and geographic locales. For instance, legumes (dried beans and peas), which are not by botanical definition vegetables, are often included for calculating vegetable intake, but not always in the same manner. Botanically speaking, foods that develop from the flower of a plant are defined as a fruit, while those from other parts are vegetables. This definition is not consistent with the culinary parlance which classifies plant-based ingredients on the basis of taste. Due to these differing approaches, inconsistencies appear between studies adding complexity to the field. Potatoes are also sometimes included in studies, but sometimes excluded. While a traditional staple for many Caucasian groups, they are not for many Asian/Pacific populations [90]. Accurate determination of vegetable consumption is essential to determine current intake patterns and for evaluating interventions developed to increase consumption. Additionally, it is not clear for the majority of the studies whether participants were asked to consider intake of vegetables per se or as part of a composite dish such as in a casserole, which may lead to inconsistencies between study findings. 

Another issue is related to the lack of investigation regarding the degree of processing of the vegetables consumed. Vegetables eaten fresh or minimally processed (i.e., unprocessed foods altered by processes to make their preparation easier or more diverse, such as removal of inedible or unwanted parts, drying, fractioning and refrigeration or placing in containers) tend to preserve their main characteristics and nutrient profile. Canned and bottled vegetables (i.e., processed foods), despite having increased durability and enhanced sensory characteristics, frequently have the addition of salt, sugar or fat as preservers, which may have a negative impact on the nutrient profile of the original food. Finally, if substances such as colours, flavours, emulsifiers and other additives are added to increase palatability and attractiveness, which is frequently done, it can be named as an ultra-processed food, whose formulation, presentation and marketing often promote overconsumption [91,92]. Differently from what is expected with fresh or minimally processed food consumption, the intake of processed, and mainly of ultra-processed, versions has been increasingly associated with unhealthy dietary nutrient profiles and several diet-related noncommunicable diseases [92,93]. These products are also troublesome from social, cultural, economic, political and environmental points of view [94]. Therefore, processed, and mainly, ultra-processed vegetables should be accounted for and analysed separately by the studies. 

Furthermore, the type of production and commercialisation of the vegetables can also have an impact on consumption. For instance, organically produced foods have higher concentrations of antioxidants, lower concentrations of the toxic metal Cd and a lower incidence of pesticide residues than non-organic comparators across regions and production seasons [95]. Additionally, they are more sustainable, with gains to species diversity in organically farmed fields in comparison to conventional farms [96]. However, organic foods are often more expensive than conventional alternatives, and this could moderate consumption [97].

It is evident that studies investigating vegetable consumption focused much more on the amount consumed than on the nutritional quality of these vegetables and the impact of their production–consumption chain. Nevertheless, such factors need to be considered, mainly when recommendations are made, as they have direct influence on human and environment health.

Studies which evaluated the frequency of vegetable intake among college students indicated a higher percentage of daily intakes in Finland, Poland, Cyprus, Lithuania and Italy. The lowest rates for daily intake were found in Saudi Arabia, South Africa and Zimbabwe. In Brazil, the frequencies of students who answered not eating vegetables at all were the highest among the studies which evaluated consumption by frequency. It would be expected that populations living within the culture of the Mediterranean diet, such as Spain, Italy, Cyprus, Croatia and Greece, at least report a higher consumption of vegetables than in other similar, non-Mediterranean, populations [98]. However, results from these countries support a shift away from the Mediterranean diet toward less healthy eating patterns [36,37,38,39,40]. This phenomenon appeared more evident among students living outside the family home, who also are more inclined towards lower consumption of homecooked meals and more frequent use of fast food [37]. Gaining primary responsibility for food shopping and preparation can lead to unhealthy dietary habits among college students living out of the family home. By contrast, students living at the family home might receive more support for healthier food habits. 

The studies which presented consumption by frequency of portions/servings consumed also indicated a more frequent consumption of 1 portion/day, which is lower than the WHO recommendation of eating at least 5 portions per day (considering vegetables and fruits together). Considering the average intake by portions, the only studies where the average reached 2 or more servings per day were in New Zealand and Canada. Additionally, the Canadian studies demonstrated high standard deviation, highlighting strong variability within these results. The terms ‘portion size’ and ‘serving size’ are sometimes used interchangeably, and therefore, both terms were included in the results. However, it is widely accepted that these terms have different meanings. Portion size refers to ‘the amount of food intended to be consumed by an individual in a single eating occasion’, whereas serving size refers to ‘the quantity recommended to be consumed in a single eating occasion’ [99]. Further, considering that not all the studies mentioned the equivalence in grams for the portions evaluated or whether participants were told what to consider as a portion, the results might not reflect exactly the same basis for assessing consumption.

The majority of studies did not mention the setting in which vegetables are consumed, which is deemed a significant gap in knowledge, especially considering that it has been previous identified that setting may have an impact on consumption, as, for instance, more frequent use of fast-food restaurants has been associated with lower intake of key nutrients and healthful foods, and conversely, more frequent use of full-service restaurants was related to higher intake of vegetables [88].

In the studies summarised according to vegetable consumption in grams/day, higher average intakes were found in Iran [35] (263 g/day) and Japan (217.5 g/days) [33]. In Iran [35], breakfast consumers had higher scores of dietary heathy eating index and dietary diversity for fruits, vegetables and whole grains compared with nonconsumers. In Japan [33], 17.5% of males and 23.5% of females had a daily vegetable intake of 350 g or more, and the results of analyses conducted separately for males and females showed that the significant relationship between breakfast skipping and poor vegetable intake was found only in males. However, it was not clear whether those who habitually eat breakfast consume vegetables at breakfast or whether they ate vegetables at other meals but not breakfast, because they did not examine dietary intake of breakfast separately. 

It is frequently stated by international agencies, national governments and nongovernmental organisations that regular breakfast consumption is associated with higher intakes of micronutrients, a better diet that includes fruits and vegetables and less frequent use of soft drinks. Further, breakfast eaters tend to achieve the recommended dietary allowance for vitamins and minerals more often compared to breakfast skippers and to have higher scores for healthy eating indexes [100]. Additionally, there is an association between skipping breakfast and low nutritional adequacy of adult diets. Moreover, comprehensive dietary counselling that supports daily breakfast consumption may be helpful in promoting healthy dietary habits throughout the day [101].

Finally, for the studies which compared vegetable consumption with relevant guidelines or recommendations, the median frequency of participants who achieved recommended vegetable intake was 35.4% but varied widely. The lowest frequency of compliance with guidelines was found in South Africa [55] and in the USA [69,84], as opposed to the highest found in Netherlands [15] and in Finland [40]. It was possible to notice, however, a wide variation in recommendations. The global guidelines from WHO do not disaggregate fruits from vegetables, and this might make it difficult for people to understand the quantities of each type of food that should be consumed. This combined recommendation hinders the standardization of national guidelines towards vegetable consumption. Irrespective of the guidelines considered, young adults are struggling to achieve the recommendation, demonstrating that this is a crucial point to be addressed.

Innovative public policy initiatives designed to stimulate vegetable consumption are required. For instance, implementing healthy food prescriptions within large government healthcare programs to promote healthier eating could generate substantial health gains and be highly cost-effective. A study using US nationally representative data and a validated microsimulation model to evaluate policy scenarios for adults found that, over a lifetime, a 30% subsidy on fruits and vegetables would prevent 1.93 million cardiovascular disease events and 0.35 million deaths and save $40 billion in healthcare costs. This subsidiary program would be highly cost-effective from a healthcare and societal perspective, and theoretical results were consistent across subgroups within each insurance group, including by age, race/ethnicity, education and income [102]. Other public health initiatives focused on influential factors identified in this study could also be discussed and implemented to increase healthy eating, such as enhancing sleeping patterns [103,104], overall wellbeing [105,106], and nutritional knowledge [34], and related to accessibility and affordability of fresh, organic and locally produced vegetables, focusing on more sustainable options [107,108] and stimulating homemade preparation [109,110]. 

### Limitations and Further Studies

Limitations are accepted on any study regarding vegetable consumption. Firstly, many studies do not disaggregate fruit from vegetables, and this is also true with regard to guidelines. Secondly, the definition of what is described as a vegetable varies considerably between studies, policy and even consumer parlance. Thirdly, measurement of vegetable intake varies from grams/day, to portions and serving sizes. Lastly, no account is taken of where vegetables are consumed or if they are part of a composite dish. The authors have ensured robust analysis of the available papers and collated key issues of debate. 

Taken together, the limitations highlight the need for greater consistency in future studies in this area. Further studies would usefully include greater consideration of the cooking method employed and the impact of this on both the nutrient contribution and consumer acceptance. Additional research into the most effective methods to encourage greater consumption levels would be valuable to both the field and this population. 

It is apparent that studies on vegetable consumption are focused on assessing consumption and the achievement of nutritional guidelines by individuals. However, it is important to think that, beyond achieving quantities, it is necessary to discuss the quality of food consumed from a health and sustainability perspective. The type of production (e.g., organic, genetically modified) and commercialisation (e.g., fair trade, local) of the vegetables are key elements to be considered, as both have impact on the sustainability of the system. The contribution of vegetables is important not just from a dietary perspective but given their potential to reflect UN sustainable development goals and deliver economic gain.

## 5. Conclusions

There is a paucity of data on the factors influencing vegetable consumption. While there have been studies of perceptions of freshness, psychosocial, environmental and life course factors influencing fruit consumption, there are very little data on vegetables, and they constitute an under-researched area. Hence, this review makes a vital and timely contribution. It is well known that the majority of students do not consume recommended levels of vegetables; however, existing efforts to understand the drivers of vegetable intake are fragmented, do not consider the population of young adults sufficiently and often aggregate fruit and vegetable consumption together, introducing bias. This review, for the first time, provides a comprehensive assessment of vegetable consumption and finds that being female is the strongest predictor associated with higher intake. Country or region differences were not observed; however, a number of other contributory factors have been identified, such as having a higher socioeconomic level; living in the family home; later stage of study; having a greater perception of happiness and less pressure and stress; and being more open to new experiences. Considering modifiable factors, overall elements related to healthy quality behaviour were the most prominent in the studies. 

Public policies and new strategies to encourage vegetable consumption among college students are indispensable, especially targeting subgroups with even lower intakes, such as males and those living outside the family home. Positive eating habits are established in young adulthood and involve adoption of healthy food practices, including adequate vegetable consumption. Additionally, recommendations broadly agree that a sustainable diet should be based on vegetable consumption and reduced meat consumption and should prioritize the consumption of locally produced, seasonal and organic foods, strengthening short food supply chains and decreasing environmental impact, climate change, soil degradation, gas emissions, water contamination and loss of biodiversity. Therefore, not only are positive eating patterns important to promote positive health outcomes such as the reduction of obesity and noncommunicable diseases, but also for global sustainability. 

## Figures and Tables

**Table 1:** Studies presenting the consumption of vegetables in frequencies of intake (*n* = 30).

Authors (Country, Year)	Study Design	Sample	Female (%)	Age Range	Mean Age	Assessment Instruments	Definition of Vegetables	Category of Results		Frequency (%)						Factors Associated with Increased Intake (*p* < 0.05)
									>1 Day ^a^	Daily ^b^	5–6 Times Per Week ^c^	3–4 Times Per Week ^d^	1–2 Times Per Week ^e^	Rarely ^f^	Never ^g^	
El Ansari et al. [47] (Finland, 2015)	Cross-sectional	1189	70.6	-	21.0	FFQ	Salad and raw vegetables, cooked vegetables	Moderate adherence to dietary guidelines (>50%)	17.6	50.9	26.8		4.6		0.2	Importance given for eating healthy
								Low adherence to dietary guidelines (<50%)	5.2	23.4	43.1		24.1		4.1	
El Ansari et al. [45] (Finland, 2015)	Cross-sectional	1189	70.6	-	21.0	FFQ	Salad and raw vegetables	Male		57.3		-	-	-	-	-
								Female		72.4		-	-	-	-	
Kowalcze et al. [75] (Poland, 2016)	Cross-sectional	100	100.0	-	-	Questionnaire	Vegetables	General		65.0		22.0	-	-	13.0	-
Hadjimbei et al. [38] (Cyprus, 2016)	Cross-sectional	193	54.9	18 to 25	20.6	Questionnaire	Fresh and cooked vegetables	General	(29.5)	56.5		-	-	-	-	-
Daniuseviciute-Brazaite & Abromaitiene [49] (Lithuania, 2018)	Cross-sectional	500	67.8	-	23.2	FFQ	Salad and raw vegetables, cooked vegetables	Cooked	5.0	60.0		40.0		20.0	0	Females
								Raw and salad	5.0	40.0		40.0		10.0	5.0	
Ramsay et al. [20] (United States, 2017)	Retrospective survey	676	63.0	18 to 25	20.8	Questionnaire	Vegetables	General	15.0	30.0		29.0	21.0	4.0	1.0	-
Teleman et al. [82] (Italy, 2015)	Cross-sectional	8516	67.0	18 to 30	22.2	Questionnaire	Vegetables	General	22.5	19.6	16.0	28.5 *	9.5	-	3.9	-
Schnettler et al. [57] (Chile, 2015)	Cross-sectional	369	53.7	-	20.9	FFQ	Vegetables	General		47.0		36.0 *	9.0	6.0	2.0	-
Khan et al. [74] (Malaysia, 2011)	Cross-sectional	460	52.8	-	21.6	Questionnaire	Vegetables	General		46.1	-	29.6	20.7	-	3.7	-
								Male		57.5	-	-	-	-	-	
								Female		42.5	-	-	-	-	-	
Hakim et al. [61] (Malaysia, 2012)	Cross-sectional	200	55.0	18 to 26	20.0	24-h recall	Vegetables	Male		43.3	45.6		4.4	4.4	2.2	-
								Female		41.8	44.5		4.5	3.6	5.5	
Likus et al. [76] (Poland, 2013)	Cross-sectional	239	84.0	-	20.0	Questionnaire	Vegetables	General		-		-	-	-	-	-
								Male		30.0	42.5		17.5	7.5	2.5	
								Female		36.7	31.6		21.2	4.5	6.0	
Evagelou et al. [36] (Greece, 2014)	Cross-sectional	435	83.4	-	-	Questionnaire	Vegetables	General		39.2	12.8	24.4	16.7	4.5	-	Females
Becerra-Bulla et al. [48] (Colombia, 2015)	Cross-sectional	45	77.2	18 to 30	-	FFQ	Vegetables without tubers, roots and bananas	General		33.3		42.2 *	15.6	-	-	-
Mushonga et al. [55] (Zimbabwe, 2013)	Cross-sectional	84	56.0	-	22.2	FFQ	Vegetables	General		14.3			9.5	17.9	2.4	-
Sousa et al. [79] (Brazil, 2014)	Cross-sectional	1232	54.7	17 to 52	23.5	Questionnaire	Vegetables	General		43.1 (≥5 days)		56.9 (≤4 days)				Regular health self-rate
van den Berg et al. [59] (South Africa, 2012)	Cross-sectional	161	68.3	18 to 42	24.9	FFQ + three 24-h recalls	Vegetables	General		12.4		-	-	85.4	2.5	-
Durán-Agüero et al. [67] (Chile, 2014)	Cross-sectional	239	23.5	18 to 31	21.5	Questionnaire	Vegetables	General	21.8		-	-	-	-	-	-
								Male		10.2	-	-	-	83.8	6.0	
								Female		17.5	-	-	-	80.8	1.7	
Ramalho et al. [30] (Brazil, 2012)	Cross-sectional	863	61.8	Grouped as ≤20 to ≥31	-	Questionnaire	Vegetables	General		40.0		-	-	-	-	-
								Male		32.7		-	-	-	-	
								Female		44.6		-	-	-	-	
Abdel- Megeid et al. [84] (Saudi Arabia, 2011)	Cross-sectional	312	57.7	-	21.1	Questionnaire + 3 days food records	Vegetables	Male		29.6	-	30.3	30.3	9.8	-	Lower BMI and lower blood pressure (both genders)
								Female		23.9	-	29.2	30.3	16.6	-	
Hilger et al. [72] (Germany, 2017)	Cross-sectional	689	69.5	-	22.7	Data from the Nutrition and Physical Activity Study	Salad and raw vegetables, cooked vegetables	Cooked	3.2	50.0		-	-	-	-	Years of university
								Raw and salad	3.6	40.0		-	38.0	-	-	-
El Ansari et al. [43] (England, Wales, Northern Ireland, 2014)	Cross-sectional	3706	72.8	-	-	FFQ	Salad and raw vegetables, cooked vegetables	Cooked vegetables			3.4 **					Less perceived stress and depressive symptoms scores (both genders)
								Salad and raw vegetables		3.6 **						
Cansian et al. [21] (Brazil, 2012)	Cross-sectional	122	94.0	-	21.0	FFQ	Sautéed leafy greens, leafy greens, vegetables	Sautéed leafy greens		-		-	38.7	9.2	25.2	-
								Leafy greens		51.2		-	33.1	1.7	7.4	
								Nonleafy cooked		15.0		-	38.4	-	15.0	
								Nonleafy raw	14.3	31.1		-	36.0	-	3.4	
Viljoen et al. [60] (South Africa, 2018)	Cross-sectional	488	44.6	18 to 24+		FFQ	Green vegetables ^1^, Yellow vegetables ^2^, Other vegetables ^3^, Salads ^4^	Green vegetables		24.6		48.5	12.7	7.3	6.9	-
								Yellow vegetables		18.1		46.8	19.2	10.0	5.8	
								Other vegetables		21.7		57.1	15.0	4.2	2.1	
								Salads		21.5		49.6	14.0	8.6	6.3	
Oliveira et al. [87] (Brazil, 2014)	Cross-sectional	97	53.6	18 to 25	-	FFQ	Vegetables, greens	Vegetables		33.0		-	-	-	-	-
								Greens		22.0		-	-	-	-	
Perez et al. [56] (Brazil, 2016)	Cross-sectional	1336	56.4	18 to 24	-	FFQ	Raw vegetables, cooked vegetables	Vegetables		21.2	41.7	-	-	-	11.4	Non-quota students
								Raw and salad		11.5	25.0	-	-	-	28.4	
								Cooked		9.5	20.8	-	-	-	25.5	
								Quota students		39.2		-	-	-	-	
								Nonquota students		43.3		-	-	-	-	
El Ansari et al. [44] (Germany, Denmark, Poland, Bulgaria, 2012)	Cross-sectional	2402	61.5	20 to 23 (70–80%)	-	FFQ	Raw and cooked vegetables	Germany	25.9			-	-	-	-	Living at family home with parents
								Denmark	19.3			-	-	-	-	
								Poland	15.2			-	-	-	-	
								Bulgaria	31.6			-	-	-	-	
Khalid et al. [24] (Pakistan, 2011)	Cross-sectional	80	100.0	-	-	Questionnaire	Vegetables	College A		50		32.5	-	-	-	-
								College B		25		55.0	-	-	-	
Juríková et al. [29] (Slovakia, 2016)	Cross-sectional	242	82.6	19 to 22	-	Questionnaire	Fresh vegetables	Journalism	22.2	22.2		-	33.3	11.1	11.1	-
								Regional Tourism in Slovakian	40.3	26.4		-	26.4	6.9	0	
								Regional Tourism in Hungarian	20.0	35.6		-	37.8	4.4	2.2	
								Education in Slovakian	18.0	37.0		-	38.0	5.0	2.0	
								Education in Hungarian	21.9	50.0		-	21.9	6.3	0	
Chen et al. [81] (Malaysia, 2018)	Cross-sectional	303	72.6	-	20.0	Questionnaire	Vegetables	General		23.4	42.9		-	33.7	-	-
								Obese		24.1	42.1		-	33.8	-	
								Non-obese		21.8	44.8		-	33.3	-	
Al-Rethaiaa et al. [64] (Saudi Arabia, 2010)	Cross-sectional	357	0.0	18 to 24	20.4	Questionnaire	Green, red or yellow coloured vegetables	General		11.2	-	24.4	32.2	32.2	-	-

FFQ: Food frequency questionnaire. a. Several times per day; b. every day; c. many times a week; d. several times a week; e. once or twice in two week/weekly; f. 1–4 times a month/several times a month/monthly/occasionally/once a week; g. do not eat. 1. broccoli, green beans, cabbage, peas, spinach; 2. butternut, carrots, pumpkin; 3. potato, cauliflower, mushroom, onions, sweet potato, mealies; 4. beetroot, lettuce, cucumber, tomatoes, sweet pepper. * 2–3 times per week. ** Refers to the mean of the consumption frequency scale referred by most participants, where 3 is ‘several times a week’ and 4 is ‘daily’.

**Table 2:** Studies presenting the consumption of vegetables in frequencies according to the portions/servings consumed (*n* = 13).

Authors	Study Design	Sample	Female (%)	Age Range	Mean Age	Assessment Instruments	Definition of Vegetables	Measure	Frequency	Factors Associated with Increased Intake (*p* < 0.05)
Anandhasayanam et al. [65] (India, 2015)	Cross-sectional	232	55.6	18 to 31	-	Questionnaire	Vegetables	<1 serving/day	5.17%,	
								1 serving/day	43.97%,	
								2 servings/day	32.76%,	
								3 servings/day	15.95%,	
								>3 servings/day	2.16%	
Crovetto et al. [66] (Chile, 2018)	Cross-sectional	1454	77.9	-	-	Questionnaire	Vegetables	Do not consume	3.3%.	Consuming two or more servings/day of vegetables was protective for overweight/obesity
								<1 portion/day	16.1%.	
								1–2 portion/day	16.8%.	
								1 portion/day	35.8%.	
								2 portions/day	27.7%	
Lesani et al. [25] (Iran, 2016)	Cross-sectional	541	75.4	-	24.1	Questionnaire	Fresh or canned or juice	<1 serving/day	22.7%	Measure of happiness was positively associated with the amount of vegetable consumption
								1 serving/day	51.6%	
								2–3 servings/day	21.1%,	
								3 servings/day	4.6%	
Durán-Agüero et al. [67] (Chile, 2014)	Cross-sectional	239	23.5	18 to 31	21.5	Questionnaire	Vegetables	2 servings/day	21.8%	
Durán-Agüero et al. [69] (Chile, 2016)	Cross-sectional	635	86.4	-	22	Questionnaire	Vegetables	Normal weight (2 portions/day)	32.4%	
								Overweight/obese (2 portions/day)	43.9%	
Feitosa et al. [70] (Brazil, 2010)	Cross-sectional	718	50.0	-	-	Questionnaire	Vegetables	<5 spoons/day	84.4%	
Poscia et al. [28] (Italy, 2017)	Cross-sectional	8292	67.0	18 to 30	22.2	Questionnaire	Vegetables	Males (>1 portion/day)	12.2%	Number of meals, breakfast intake (stronger for males)
								Females (>1 portion/day)	27.5%	
Ilow et al. [73] (Poland, 2017)	Cross-sectional	1168	76.4		23.5	Questionnaire with 46 questions	Vegetable	Male (≥3 portions/day)	21.0%	
								Female (≥3 portions/day)	30.2%	
Sharma et al. [78] (Germany, 2009)	Cross-sectional	305	63.3	18 to 24	-	Questionnaire	Vegetables	Total (<1 serving/day)	8.3%	Living at family home
								Dependent	4.8%	
								Independent	9.6%.	
								Total (1 serving/day)	23.2%	
								Dependent	25.3%	
								Independent	22.5%.	
								Total (2 servings/day)	32.1%	
								Dependent	37.3%	
								Independent	29.8%.	
								Total (3 servings/day)	36.8%	
								Dependent	20.5%	
								Independent	23.9%	
								Total (4–5 servings/day)	13.6%	
								Dependent	12.0%	
								Independent	14.2%	
Yahia et al. [83] (United States, 2016)	Cross-sectional	237	73.0	-	20.6	Questionnaire	Vegetables	Male (≥2 portions/day) Always	28.0%	
								Often	16.0%	
								Sometimes	45.0%	
								Never	11.0%	
								Female (≥2 portions/day) Always	22.0%	
								Often	36.0%,	
								Sometimes	32.0%,	
								Never	10.0%	
Doostan et al. [50] (Iran, 2016)	Cross-sectional	229	66.0	-	21.8	FFQ	Vegetables	Categories of Healthy Eating Index: Poor (2.83 cups/day)	8.1%	-
								Needs improvement (2.89 cups/day)	63.4%	
								Good (3.18 cups/day)	28.5%	
Gresse et al. [51] (South Africa, 2015)	Cross-sectional	619	66.1	-	-	FFQ	Vegetable	Health area (<1 portion/day)	70.0%	
								Other areas (<1 portion/day)	64.0%	
El Hamid Hussein [23] (Saudi Arabia, 2011)	Cross-sectional	205	100.0	18 to 21	-	FFQ	Vegetable	Nutrition department students (≥3 servings/day)	64.3%	
								Non-nutrition department students (≥3 servings/day)	45.5%	

FFQ: Food frequency questionnaire.

**Table 3:** Studies presenting the average servings per day of vegetables consumed overall or by groups (*n* = 13).

Authors	Study Design	Sample	Female (%)	Age Range	Mean Age	Assessment Instruments	Definition of Vegetables	Measure (Servings/Day)	Mean (SD)	Factors Associated with Increased Intake (*p* < 0.05)
Bagordo et al. [37] (Italy, 2013)	Cross-sectional	193	77.7	20 to 30	-	FFQ	Raw vegetables, cooked vegetables	Raw vegetables *	4.8 (≅3.00)	Living at family home higher intake of cooked vegetables. Living away from home higher intake of raw vegetables
								Cooked vegetables *	3.0 (≅2.50)	
								Living at home		
								Raw vegetables *	4.40 (3.00)	
								Cooked vegetables *	3.29 (2.56)	
								Living outside home		
								Raw vegetables *	5.74 (3.52)	
								Cooked vegetables *	2.18 (2.05)	
Lupi et al. [53] (Italy, 2015)	Cross-sectional	258	-	19 to 42	23.3	FFQ	Salad and raw vegetables, cooked vegetables	General		Females and living at family home
								Raw vegetables/salads *	4.69 (≅0.5)	
								Cooked vegetables *	3.10 (≅0.5)	
								Male		
								Raw vegetables/salad *	3.46 (≅2.87)	
								Cooked vegetables *	1.92 (2.45)	
								Female		
								Raw vegetables/salads *	5.25 (3.81)	
								Cooked vegetables *	3.63 (4.10)	
								Living at home		
								Raw vegetables *	5.78 (3.94)	
								Cooked vegetables *	3.91 (3.34)	
								Living outside home		
								Raw vegetables *	3.76 (3.08)	
								Cooked vegetables *	2.40 (3.95)	
Conner et al. [22] (New Zealand, 2017)	Cross-sectional	1,073	67.9	18 to 25	20.6	Daily diary for 21 (sample 1) or 13 days (sample 2)	Fresh, frozen, canned, or stewed vegetables. *Do not include vegetable juices or hot chips (French fries).	Sample 1	2.51 (1.07)	Openness to experience
								Sample 2	2.76 (1.38)	
De Piero et al. [19] (Argentina, 2015)	Time series analysis	329	75.0	-	23.0	FFQ + 7-days food record	Raw and cooked vegetables (portion 150 g)	General	0.7 (-)	-
								1998–1999 ^§^	0.5 (-)	
								2012–2013 ^§^	0.9 (-)	
Kresic et al. [52] (Croatia, 2009)	Cross-sectional	1005	73.7	-	21.7	FFQ + Questionnaire	Vegetables	Total	2.17 (1.27)	Nutrition knowledge
								Male	1.67 (0.83)	
								Female	1.81 (1.00)	
								Freshman	1.63 (0.71)	
								Juniors	1.83 (0.97)	
								Seniors	1.65 (0.99)	
								Home	1.98 (0.93)	
								Student restaurants	1.80 (0.97)	
								Self-cooking	1.72 (0.99)	
Muñiz-Mendoza [27] (Chile, 2018)	Cross-sectional	218	62.8	18 to 30	20.7	FFQ + Questionnaire + 24-h recall	Vegetables (not included fruits, cereals and tubers)	Male ^§^	0.47 (0.85)	
								Female ^§^	1.08 (1.34)	
								<20 years	0.82 (1.13)	
								20–25 years	0.95 (1.27)	
								>25 years	1.33 (1.73)	
Lim et al. [26] (Singapore, 2017)	Cross-sectional	884	49.3	20 to 22	-	Questionnaire	Vegetables	General	1.7 (-)	.
Menozzi et al. [33] (Italy, 2017)	Cross-sectional	751	55.0	-	22.1	Questionnaire	Vegetables	General	1.8 (-)	Consumption may be intentional as well as habitual, depending on the level of habit strengths
Muñoz de Mier et al. [54] (Spain, 2017)	Cross-sectional	390	60.0	18 to 25	21.3	FFQ	Vegetables	General ^§^	1.6	Females and normal weight
Pérez-Gallardo et al. [39] (Spain, 2015)	Cross-sectional	77	80.0	-	21.0	7-day food record	Vegetables	General ^§^	0.9 (0.5)	
								Male	1.0 (0.6)	
								Female	0.9 (0.5)	
Pérusse-Lachance et al. [77] (Canada, 2010)	Cross-sectional	2490	76.0	-	24.1	Questionnaire	Raw or cooked or canned or frozen	General	2.5 (1.6)	
Strawson et al. [40] (Canada, 2013)	Cross-sectional	36	100.0	-	-	FFQ	Vegetables	General	2.7 (1.3)	.
White et al. [18] (New Zealand, 2013)	Microlongitudinal (21 days)	281	54.4	18 to 25	19.9	21 days food diary	Vegetables	General	2.51 (-)	Greater positive affects

FFQ: Food frequency questionnaire. * Servings/week. ^§^ Used the term ‘portion’ instead of serving.

**Table 4:** Studies presenting vegetable consumption in grams/day (*n* = 8).

Authors	Study Design	Sample	Female (%)	Age Range	Mean Age	Assessment Instruments	Definition of Vegetables	General Results for Vegetable Consumption	Average Intake In Grams/Day (SD)	Factors Associated with Increased Intake (*p* < 0.05)
Azadbakht & Esmaillzadeh [41] (Iran, 2012)	Cross-sectional	289	100.0	18 to 28	-	FFQ	Vegetables	General	263.0	Lower dietary energy density
Azadbakht et al. [42] (Iran, 2013)	Cross-sectional	411	100.0	18 to 28	-	FFQ	Vegetables	Breakfast eaters	185.0	Breakfast consumption
								Breakfast skippers	133.0	
Fujii et al. [35] (Japan, 2010)	Cross-sectional	125	54.4	18 to 21	19.2	Diet history	Vegetables	General	217.5 (156.6)	
								20.8%	≥350.0	
Murakami et al. [62] (Japan, 2012)	Cross-sectional	3956	100.0	18 to 20	-	Diet history	Vegetables	General	126.7 (75.5)	
Nola et al. [86] (Croatia, 2010)	Cross-sectional	441	70.0	19 to 26	-	FFQ	Vegetables	1st year	80.0 (47.3)	
								6th year	129.0 (47.4)	
Sato-Mito et al. [63] (Japan, 2011)	Cross-sectional	3304	100.0	18 to 20	-	Self-administered diet history questionnaire	Coloured vegetables or other vegetables or mushrooms or sea vegetables	General	121.6 (71.6)	Early midpoint of sleep
								Midpoint of sleep quintile 1st	126.7 (3.1)	
								2nd	127.5 (2.6)	
								3rd	121.9 (2.8)	
								4th	121.3 (2.6)	
								5th	109.8 (2.93)	
Teschl et al. [34] (Germany, 2018)	Cross-sectional	365	86.0	17 to 53	-	FFQ	Cooked and raw vegetables	Male	179.0 (153)	
								Female	176.0 (165)	
van der Bogerd et al. [32] (The Netherlands, 2018)	Cross-sectional	717	63.7	<22>	-	FFQ	Vegetable side dishes or salads	General	126.2	Adherence to the vegetable guideline was met by 6%–8% of the students

FFQ: Food frequency questionnaire.

**Table 5:** Studies comparing the consumption of vegetables with relevant guidelines (*n* = 14).

Authors	Study Design	Sample	Female (%)	Age Range	Mean Age	Assessment Instruments	Definition of Vegetables	Guidelines	Categories	Meeting the Recommendations (%)			Factors Associated with Increased Intake (*p* < 0.05)
										General	Male	Female	
El Ansari et al. [47] (Finland, 2015)	Cross-sectional	1189	70.6	-	21.0	FFQ	Salad and raw vegetables, cooked vegetables	WHO dietary guidelines (daily or several times a day)	Salad/raw vegetables	68.4	57.6	72.9	Females and Importance given for eating healthy
									Cooked vegetables	28.6	19.8	32.3	
El Ansari et al. [46] (Finland, 2015)	Cross-sectional	1189	70.6	-	21.0	FFQ	Salad and raw vegetables, cooked vegetables	WHO dietary guidelines (daily or several times a day)	Salad/raw vegetables	-	57.2	72.9	Females and Less stress
									Cooked vegetables	-	19.8	32.6	
Becerra-Bulla et al. [48] (Colombia, 2015)	Cross-sectional	45	77.2	18 to 30	-	FFQ	Vegetables without tubers, roots and bananas	Colombian Food Guidelines (5 servings/day)		24.5	-	-	-
Strawson et al. [40] (Canada, 2013)	Cross-sectional	36	100.0	-	-	FFQ	-	Traditional Healthy Mediterranean Diet Pyramid (≥4 servings/day)		19.0			
Muñoz de Mier et al. [54] (Spain, 2017)	Cross-sectional	390	60.0	18 to 25	21.3	FFQ	-	Food Diamond—Spanish Guidelines (≥3 servings/day)		21.0	12.7	31.8	Females and normal weight
Stroebele-Benschop et al. [58] (Germany, 2018)	Cross-sectional	103	75.7	18 to 30	24.3	FFQ	Raw or cooked vegetables or salad	German Nutrition Society (≥3 servings/day)		12.9	4.2	15.4	-
Teschl et al. [34] (Germany, 2018)	Cross-sectional	365	86.0	17 to 53	-	FFQ	Cooked and raw vegetables	German Nutrition Society (≥3 servings or 400g/day)			9.8	7.3	
van den Berg et al. [59] (South Africa, 2012)	Cross-sectional	161	68.3	18 to 42	24.9	FFQ; three 24-h recalls	-	USDA Food Guide Pyramid (≥3 servings/day)		2.5	-	-	-
Greene et al. [71] (United States, 2011)	Cross-sectional	1689	72.4	18 to 19	-	Questionnaire	-	MyPyramid (2.5 cups/day)		12.4	8.9	14.4	-
McArthur & Pawlak [85] (United States, 2011)	Cross-sectional	149	75.2	-	20.9	3-days food records	Vegetables and juice	MyPyramid (2.5 cups/day)		7.0	-	-	-
Odum & Xu [31] (United States, 2018)	Cross-sectional	1503	59.1	18 to 25+	19.1	Questionnaire	Fresh, cooked, or frozen, as well as green salad and to not count potatoes	Dietary Guidelines for Americans (2.5 cups/day)		On average, in 3 of the past 7 days)	-	-	-
Durán-Agüero et al. [68] (Chile, 2015)	Cross-sectional	634	87.0	-	22.0	Questionnaire	-	Chilean Food Guidelines (≥2 servings/day)		35.4	-	-	Adequate weight
van der Kruk et al. [17] (The Netherlands, 2014)	Cross-sectional and longitudinal	568	96.0	18 to 29	-	7 days food records + questionnaire	-	Netherlands Guidelines for a Healthy Diet (≥150 g/day)	First-year students	40.0	-	-	-
									Fourth-year students	74.0	-	-	
Sousa et al. [80] (Brazil. 2013)	Cross-sectional	1084	55.0	-	23.5	Questionnaire	-	WHO dietary guidelines—adapted (≥5 days/week)	All ages	43.0	39.0	46.5	Females
									17 to 19years	43.5	-	-	
									20 to 21years	35.6	-	-	
									22 to 24years	40.3	-	-	
									25 to 52years	53.2	-	-	

FFQ: Food frequency questionnaire.
